# Grape Seed Proanthocyanidin Extract Prevents Bone Loss via Regulation of Osteoclast Differentiation, Apoptosis, and Proliferation

**DOI:** 10.3390/nu12103164

**Published:** 2020-10-16

**Authors:** Sung Chul Kwak, Yoon-Hee Cheon, Chang Hoon Lee, Hong Young Jun, Kwon-Ha Yoon, Myeung Su Lee, Ju-Young Kim

**Affiliations:** 1 Department of Anatomy, School of Medicine, Wonkwang University, Iksan 54538, Korea; ksc960@naver.com; 2 Musculoskeletal and Immune Disease Research Institute, School of Medicine, Wonkwang University, Iksan 54538, Korea; hanleuni@naver.com (Y.-H.C.); lch110@wonkwang.ac.kr (C.H.L.); 3 Division of Rheumatology, Department of Internal Medicine, Wonkwang University Hospital, Iksan 54538, Korea; 4 Medical Convergence Research Center, Wonkwang University Hospital, Iksan 54538, Korea; zip80@wku.ac.kr (H.Y.J.); khy1646@wku.ac.kr (K.-H.Y.); 5 Department of Radiology, School of Medicine, Wonkwang University, Iksan, Jeonbuk 570-749, Korea

**Keywords:** grape seed proanthocyanidin extract, osteoclast, proliferation, differentiation, apoptosis, bone disease

## Abstract

Dietary procyanidin has been shown to be an important bioactive component that regulates various pharmacological activities to maintain metabolic homeostasis. In particular, grape seed proanthocyanidin extract (GSPE) is a commercially available medicine for the treatment of venous and lymphatic dysfunction. This study aimed to investigate whether GSPE protects against lipopolysaccharide (LPS)-induced bone loss in vivo and the related mechanism of action in vitro. The administration of GSPE restored the inflammatory bone loss phenotype stimulated by acute systemic injection of LPS in vivo. GSPE strongly suppressed receptor activator of nuclear factor kappa-B ligand (RANKL)-induced osteoclast differentiation and bone resorption activity of mature osteoclasts by decreasing the RANKL-induced nuclear factor-κB transcription activity. GSPE mediates this effect through decreased phosphorylation and degradation of NF-κB inhibitor (IκB) by IκB kinaseβ, subsequently inhibiting proto-oncogene cellular Fos and nuclear factor of activated T cells. Additionally, GSPE promotes osteoclast proliferation by increasing the phosphorylation of components of the Akt and mitogen-activated protein kinase signaling pathways and it also inhibits apoptosis by decreasing the activity of caspase-8, caspase-9, and caspase-3, as corroborated by a decrease in the Terminal deoxynucleotidyl transferase dUTP nick end labeling -positive cells. Our study suggests a direct effect of GSPE on the proliferation, differentiation, and apoptosis of osteoclasts and reveals the mechanism responsible for the therapeutic potential of GSPE in osteoclast-associated bone metabolism disease.

## 1. Introduction

Bone diseases are caused by disharmony in the bone remodeling process, which is mediated by bone-formative osteoblasts and bone-resorbing osteoclasts [1]. Excessive activation of osteoclasts, which leads to excessive bone destruction, is associated with low concentration levels of estrogen in post-menopausal women or excessive inflammatory responses in pathological circumstances [2,3].

Osteoclasts are derived from bone-marrow macrophages and are responsible for the removal of old bone tissue by secreting acid and collagenase [4,5]. Osteoclastogenesis needs two factors, macrophage colony-stimulating factor (M-CSF) for proliferation and survival, and receptor activator of nuclear factor kappa-Β ligand (RANKL) for differentiation and function [5]. M-CSF has a crucial role in sustaining the survival and proliferation of osteoclast precursor cells [6]. The binding of M-CSF to c-Fms on the osteoclast cell surface leads to the recruitment of c-Cbl and phosphatidylinositol 3-kinase (PI3K) complex. This complex activates the Akt pathway and causes c-Fms ubiquitination [7], which results in an increase in tyrosine phosphorylation and activation. Besides, M-CSF augments Grb2, which mediates the phosphorylation of ERK [8]. Thus, the M-CSF-induced activation of c-Fms plays a crucial role in the proliferation and survival of osteoclast precursor cells through the PI3K/Akt and ERK pathways [7,8,9]. The binding of RANKL to its cognate receptor RANK results in the activation of signaling pathways, including components of the mitogen-activated protein kinase (MAPK) pathway, such as c-Jun N-terminal protein kinase (JNK), p38, and ERK through tumor necrosis factor receptor-associated factor 6 (TRAF6) [9]. It has also been reported that the JNK signaling pathway plays an important role in the regulation of apoptosis [10]. Nuclear factor-κB (NF-κB) is one of the key transcription factors for osteoclast differentiation, which is activated by RANKL. RANKL phosphorylates NF-κB inhibitor (IκB), then activates ubiquitin-dependent proteasomal degradation of IκB, resulting in activation of transcription after NF-κB is translocated from the cytoplasm to the nucleus [11,12]. Activation of the TRAF6 family stimulates the auto-amplification of nuclear factor of activated T cells (NFAT)c1, the key factor for osteoclastogenesis [13], which induces the expression of osteoclast-specific marker genes, such as osteoclast-associated receptor (OSCAR), cathepsin K (CtsK), and tartrate-resistant acid phosphatase (TRAP).

Grape seed proanthocyanidin extract (GSPE), which is extracted from European red grape seed *Vitis vinifera*, is a commercially available medicine for the treatment of venous and lymphatic dysfunction [14,15]. Grapes have healthful properties with ingredients like phenolic bioflavonoids that are powerful antioxidants [15,16]. Related studies on the effects of GSPE in animals have demonstrated the potent anti-inflammatory properties of proanthocyanidins and their impact on experimental inflammation [17]. Recent studies on GSPE have also reported its protective effects on monosodium iodoacetate-induced arthritis and collagen-induced arthritis [18,19,20]. However, the effects of GSPE on osteoclast proliferation, differentiation, and apoptosis in bone metabolism have not yet been researched.

We investigated whether GSPE protects against lipopolysaccharide (LPS)-induced bone loss in mouse to identify whether it could be used as a candidate medicine for the possible treatment of bone metabolic disease. We also studied its effects on the proliferation, differentiation, and apoptosis of osteoclasts and the related mechanism of action in vitro.

## 2. Materials and Methods

### 2.1. Mice, Reagents and Antibodies

Five-week old male ICR mice were purchased from Samtako Bio Korea (Osan, Korea). The mice were kept in a temperature-controlled (22–24 °C) and humidity-controlled (55–60%) environment with a 12 h light/dark cycle. All experiments were performed in accordance with the guidelines of the Institute Committee of Wonkwang University for animal experimentation (Approval Number: WKU20-39). GSPE was obtained from the Hanlim pharmaceutical company (Seoul, Korea), and was prepared as a stock solution of 25 mg/mL in distilled water (DW) and stored at −20 °C. Anti-β-actin monoclonal antibody and LPS were obtained from Sigma (St. Louis, MO, USA). LPS was dissolved in PBS at 5 mg/mL and stored it at −20 °C. Soluble, recombinant human M-CSF and human RANKL were obtained from PeproTech EC Ltd. (London, UK). Anti-p38, anti-phospho-p38, anti-JNK, anti-phospho-JNK, anti-Akt, anti-phospho-Akt, anti-phospho-ERK, anti-ERK, anti-PLCγ2, anti-phospho-PLCγ2, anti-Btk, anti-IκB, and anti-phospho-IκB antibodies were purchased from Cell Signaling Technology Inc. (Beverly, MA, USA). Anti-phospho-Btk was purchased from GeneTex (Irvine, CA, USA). Anti-c-Fos and anti-NFATc1 antibodies were purchased from Santa Cruz Biotechnology (Santa Cruz, CA, USA). Secondary antibodies, including horseradish peroxidase-conjugated anti-mouse IgG and anti-rabbit IgG were obtained from Enzo Life Sciences (Farmingdale, NY, USA). Fetal bovine serum (FBS), α-minimum essential medium (α-MEM), and penicillin/streptomycin antibiotics were purchased from Gibco BRL (Grand Island, NY, USA). All other chemicals were of analytical grade or complied with the standards required for cell culture.

### 2.2. In Vitro Osteoclastogenesis Assay

Bone marrow cells (BMCs) were obtained from 5-week old male ICR mice by flushing the femurs and tibias with α-MEM supplemented with 10% FBS and 1% antibiotics. To obtain bone marrow macrophages (BMMs), BMCs were seeded on culture dishes in α-MEM supplemented with 10% FBS and 10 ng/mL M-CSF and cultured for 1 day. Non-adherent cells were transferred to 10 cm petri dishes and further cultured in the presence of 30 ng/mL M-CSF for 3 days. After removal of the non-adherent cells, the adherent cells were used as the osteoclast precursors. To generate osteoclasts from these osteoclast precursors, the cells were seeded in a 48-well plate (3.5 × 10^4^ cells/well) in complete medium, containing 30 ng/mL M-CSF and 100 ng/mL RANKL, and cultured for 4 days with or without GSPE. The cells were fixed in 3.7% formalin for 10 min, permeabilized with 0.1% Triton X-100 (Sigma, St. Louis, MO, USA), and then stained with TRAP (Sigma). TRAP-positive multinucleated cells with more than 3 nuclei were counted as osteoclasts.

### 2.3. Fluorescence Microscopy Analysis

BMMs were seeded in a 24-well plate (3.5 × 10^4^ cells/well) in complete medium containing 30 ng/mL M-CSF and cultured for 48 h with or without GSPE. The cells were fixed in 3.7% formalin for 10 min, permeabilized with 0.1% Triton X-100, and then stained with Terminal deoxynucleotidyl transferase dUTP nick end labeling (TUNEL) using the ApopTag^®^ Fluorescein In Situ Apoptosis Detection kit (Millipore, Darmstadt, Germany).

### 2.4. Proliferation Assay

XTT assay was performed to examine the effects of GSPE on the proliferation of osteoclast precursors. BMMs were seeded into 96-well plates at a density of 1 × 10^4^ cells/well with various concentrations of GSPE and incubated for 3 days in the presence of 30 ng/mL M-CSF. Following that, 50 μL XTT solution was added to each well and incubated for 4 h. The plate was read at 450 nm using an ELISA reader (Molecular Devices, Sunnyvale, CA, USA).

### 2.5. Pit Formation Assay

For the assay, 1 × 10^7^ BMCs cells and 1 × 10^6^ primary osteoblast cells were seeded on collagen gel-coated culture dishes and cultured for 9 days in the presence of 10 nM 1,25-dihydroxyvitamin D_3_ (Sigma) and 1 μM prostaglandin E_2_ (PGE_2_) (Sigma). The co-cultured cells were detached by treatment with 0.1% collagenase at 37 °C for 10 min and then re-plated onto hydroxyapatite (HA)-coated plates (Corning Inc., Corning, NY, USA) and dentin slices. The cells were incubated on the plates with or without GSPE. After 24 and 48 h, the cells were removed using 10% sodium hypochlorite and the total resorption pits were photographed and analyzed using Image-Pro Plus version 4.0 (Media Cybernetics, Silver Spring, MD, USA).

### 2.6. Gene Expression Analysis Using Quantitative Real-Time Reverse Transcription Polymerase Chain Reaction (qRT-PCR)

Total RNA was extracted from cells using Trizol reagent (Invitrogen, Carlsbad, CA, USA), and the cDNA was synthesized at 42 °C for 1 h using 1 μg RNA, oligo (dT), and an M-MLV RT kit (Invitrogen). Real-time RT-PCR was carried out on an Exicycler 96 Real-Time Quantitative Thermal Block (Bioneer Co., Daejeon, Korea) in a 20 μL reaction mixture containing 10 μL of SYBR^®^ Green Premix (Bioneer Co.), 1 μL of each primer (10 pmol), and 1 μL of cDNA. Primer sets used are shown in Table 1. The specificity of the SYBR green assays was confirmed using melting-point analysis. The mRNA levels of genes were calculated from the cycle threshold (Ct) value using the 2(-Delta pool Ct) method [21]. All reactions were run in triplicate and the data were normalized to *glyceraldehyde-3-phosphate dehydrogenase (GAPDH)* levels.

### 2.7. Western Blot Analyses

Whole-cell lysates were prepared using lysis buffer containing 50 mM Tris-HCl, 150 mM NaCl, 5 mM EDTA, 1% Triton X-100, 1 mM sodium fluoride, 1 mM sodium vanadate, 1% deoxycholate, and protease inhibitors. Whole-cell lysates was centrifuged at 13,500× *g* for 15 min, and then the supernatant was collected. The protein concentration was measured using a DC Protein Assay kit (Bio-Rad Laboratories Inc., Hercules, CA, USA). Equal amounts of protein (20–30 µg) were run on 10% SDS-PAGE gels and transferred by electroblotting onto polyvinylidene difluoride membranes (Millipore, Bedford, MA, USA). The membranes were blocked with 5% nonfat milk in Tris-buffered saline containing 0.1% Tween-20 (TBST) for 1 h, followed by blotting with the primary antibodies for 2 h at room temperature. After washing the membranes with TBST, they were incubated for 1 h with horseradish peroxidase-conjugated sheep anti-mouse or donkey anti-rabbit immunoglobulin antibodies. The specific signals were detected using the Western Blotting Detection Reagent kit (Millipore).

### 2.8. Luciferase Assay

293R cells were plated in 24-well plates as triplicates for each condition and then transfected with differing amounts of the following cDNAs: NF-kB luciferase and β-galactosidase. After 48 h, the transfected cells were lysed using Cell Culture Lysis Reagent (Promega, Madison, WI, USA), and luciferase activity was measured using the Luciferase Assay System from Promega. Luciferase activity was normalized to the β-galactosidase activity in each sample.

### 2.9. Retrovirus Preparation and Infection

Packaging of the retroviral vectors pMX-IRES-EGFP and pMX-constitutively active (CA)-Iκκβ-IRES-EGFP were transfected by transient transfection of these pMX vectors into Plat-E retroviral packaging cells using X-tremeGENE 9 (Roche, Nutley, NJ, USA), according to the manufacturer’s protocol. After incubation in fresh medium for 2 days, the culture supernatants of the retrovirus-producing cells were collected. For retroviral infection, non-adherent BMCs were cultured in 30 ng/mL M-CSF. After 2 days incubation, the BMMs were infected with the viral supernatants of the pMX-IRES-EGFP and pMX-CA-Iκκβ-IRES-EGFP virus-producing Plat-E cells together with 10 mg/mL polybrene and 30 ng/mL M-CSF for 6 h. The infection efficiency of the retroviruses was determined by green fluorescent protein (GFP) expression and was always found to be greater than 80%. After infection, the BMMs were induced to differentiate in the presence of 30 ng/mL M-CSF and 100 ng/mL RANKL for 4 days. Osteoclast formation was detected using TRAP staining.

### 2.10. Model of LPS-Induced Bone Loss and Treatment

To study the effect of GSPE on LPS-induced osteoclast formation in vivo, ICR mice were divided into four experimental groups composed of 5 mice each: PBS-treated (control), only LPS-treated, only GSPE-treated, and LPS+GSPE-treated groups. Further, 200 mg/kg GSPE or PBS was injected intraperitoneally 1 day before the LPS (5 mg/kg) injection. GSPE or PBS was administered orally every other day for 8 days. LPS was injected intraperitoneally on days 1 and 4. The mice were euthanized after 8 days and the left and right femurs were analyzed using high-resolution micro-computed tomography (micro-CT). The femur metaphysic regions were scanned using high-resolution micro-CT (NFR-Polaris-S160; Nanofocus Ray, Iksan, Korea) with a source voltage of 55 kVp, current of 60 µA, and 6 µm isotropic resolution. Femur scans were performed from the growth plate until 2 mm proximally, with 720 sections per scan. Bone histomorphometric analyses were performed with the micro-CT data using INFINITT-Xelis software (INFINITT Healthcare, Seoul, Korea). The structural parameters analyzed included trabecular bone volume/total volume (BV/TV, %), trabecular thickness (Tb.Th, μm), trabecular separation (Tb.Sp, μm), and trabecular number (Tb.N, 1/mm). The femurs were fixed in 4% paraformaldehyde (Sigma) for 1 day, decalcified for 2 weeks in 12% EDTA, and then embedded in paraffin. Sections (5 µm thick) were prepared using Leica microtome RM2145 (Leica Microsystems, Bannockburn, IL, USA) and stained with hematoxylin and eosin (H&E) and TRAP. Representative images were captured using a light microscope and the number of osteoclasts per field was counted using Image Pro-Plus software version 4.0 (Media Cybernetics, Silver Spring, MD, USA).

### 2.11. Statistical Analyses

Experiments were conducted at least three times and the data are expressed as mean ± standard deviation or mean ± standard error. All statistical analyses were performed using GraphPad Prism 8 (GraphPad Software, San Diego, CA, USA). Student’s *t*-test was used to compare the parameters between 2 groups, while the analysis of variance (ANOVA) test followed by the Tukey post-hoc test was used to compare the parameters among 3 groups. Data with *p* values < 0.05 were considered statistically significant.

## 3. Results

### 3.1. GSPE Prevents LPS-Induced Inflammatory Bone Loss by Attenuating Osteoclast Formation In Vivo

**Figure 1:**
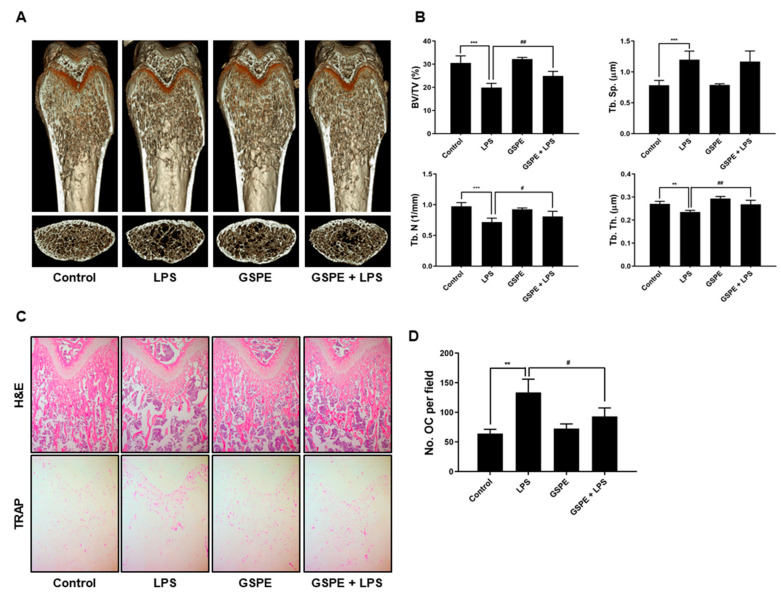
GSPE inhibits LPS-induced inflammatory bone loss in vivo. (**A**) GSPE (200 mg/kg) or PBS was administered orally on the day before LPS injection (5 mg/kg) to mice. LPS was injected intraperitoneally on days 1 and 4. GSPE or PBS was administered orally every other day for 8 days. Mice were euthanized 8 days after the first LPS injection and radiographs of the longitudinal and transverse sections of the proximal femur were obtained using a micro-CT apparatus. (**B**) The BV/TV, Tb.Sp, Tb.Th, and Tb.N of the femurs were determined by analyzing the micro-CT data using INFINITT-Xelis. (**C**) Dissected femurs were fixed, decalcified, paraffin embedded, and sectioned. Sections were stained with H&E and TRAP. The pink area stained by TRAP represents osteoclasts (OCs). (**D**) Number of osteoclasts per field of tissue was analyzed using the histomorphometric results. The OC numbers per field are the representative of three independent experiments, and 5 files per slide were quantified. ** *p* < 0.01; *** *p* < 0.001 versus the control group, *^#^ p* < 0.05; *^##^ p* < 0.01 versus the LPS group. Abbreviations: GSPE, grape seed proanthocyanidin extract; LPS, lipopolysaccharide; H&E, hematoxylin and eosin; BV/TV, trabecular bone volume/total volume; Tb.Th, trabecular thickness; Tb.Sp, trabecular separation; Tb.N, trabecular number; TRAP, tartrate-resistant acid phosphatase; OCs, osteoclasts.

To investigate the effect of GSPE on LPS-induced inflammatory bone loss, we performed high-resolution micro-CT and histology of femurs from mice treated with GSPE or vehicle, following injection of LPS or PBS. Compared to PBS alone, LPS induced significant bone loss with a decrease in BV/TV, Tb.Th, and Tb.N and an increase in Tb.Sp (Figure 1A,B). However, administration of GSPE with LPS attenuated the LPS-induced bone loss. Treatment with GSPE+LPS increased the BV/TV, Tb.Th, and Tb.N. compared to LPS alone; on the other hand, GSPE alone did not exhibit any significant difference compared to the control (Figure 1A,B). The H&E stained histological sections of the femur also showed the protective effect of GSPE on LPS-induced trabecular bone loss (Figure 1C). The LPS-induced group showed increased inflammatory osteolysis compared to the control, and the bone matrix in the LPS-induced group treated with GSPE was restored. As shown in Figure 1C, lower and 1D, osteoclast numbers per field (as assessed using in vivo TRAP staining) were also significantly reduced when GSPE was administrated to the LPS-treated mice, indicating that GSPE reduced the number of osteoclasts in LPS-treated mice. Based on these observations, we concluded that GSPE inhibits osteoclast formation, thereby inhibiting bone resorption in vivo.

### 3.2. GSPE Inhibits RANKL-Induced Osteoclast Differentiation and Bone Resorption In Vitro

**Figure 2:**
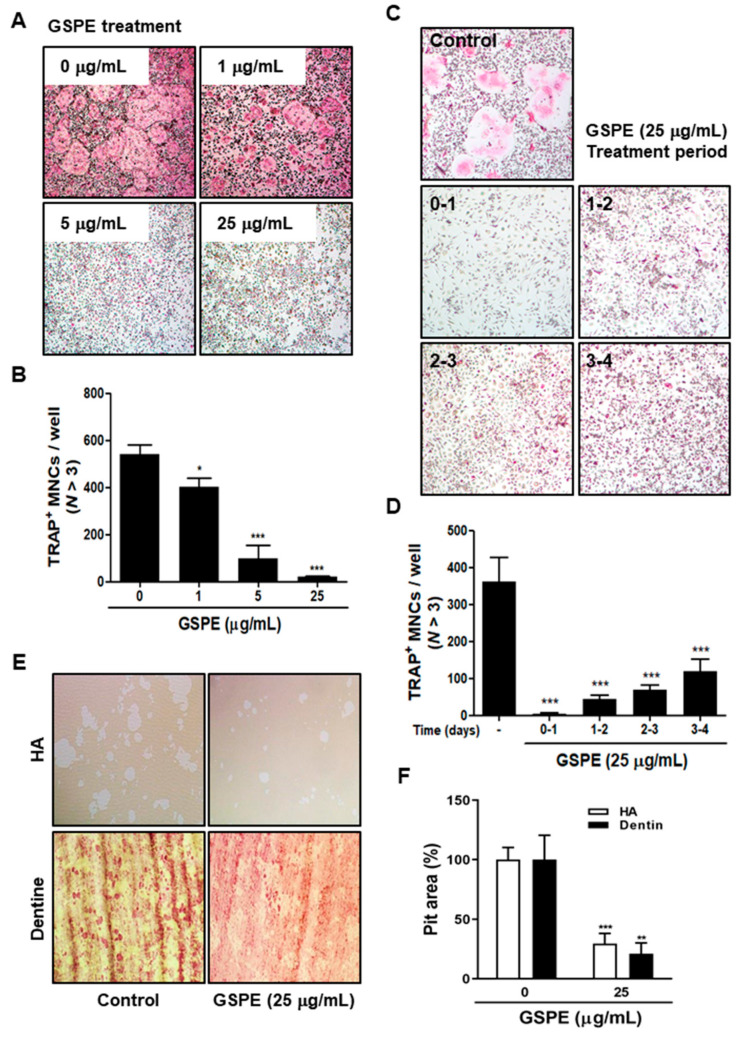
GSPE represses RANK-induced osteoclast differentiation and bone resorption activity. (**A**) BMMs were cultured for 4 days in the presence of 30 ng/mL M-CSF and 100 ng/mL RANKL in DW or GSPE. Cells were fixed with 3.7% formalin, permeabilized with 0.1% Triton X-100, and stained with TRAP solution. (**B**) TRAP-positive multinucleated cells were counted as osteoclasts. (**C**) BMMs were cultured as in A, and in addition treated with 25 μg/mL GSPE on the indicated days. After culturing, cells were stained and (**D**) the number of TRAP-positive MNCs was counted. (**E**) Mature osteoclasts were seeded on HA-coated plates or dentin slices and treated for 12 or 48 h with 25 μg/mL GSPE. Attached cells on the plates were removed and photographed under a light microscope. (**F**) Pit areas were quantified using ImageJ. * *p* < 0.05; ** *p* < 0.01; *** *p* < 0.001 versus control. Abbreviations: GSPE, grape seed proanthocyanidin extract; RANKL, receptor activator of nuclear factor kappa-Β ligand; BMMs, bone marrow macrophages; M-CSF, macrophage colony-stimulating factor; DW, distilled water; TRAP, tartrate-resistant acid phosphatase; MNCs, multinucleated cells; HA, hydroxyapaptite.

To examine whether GSPE affects the RANKL-induced osteoclast differentiation, BMMs were incubated with M-CSF and RANKL in the presence of various concentrations (0, 1, 5, 25 μg/mL) of GSPE. As seen in Figure 2A,B, GSPE resulted in a concentration-dependent inhibition of RANKL-induced osteoclast differentiation, and 1, 5, and 25 μg/mL of GSPE displayed inhibitory percentages of 25.6%, 81.6%, and 96.2%, respectively. GSPE exhibited no cytotoxicity in BMMs, indicating that its anti-osteoclastogenesis was not due to cytotoxicity (Figure S1). To confirm the time point of action of GSPE on the inhibition of osteoclastogenesis, BMMs were treated with 25 μg/mL GSPE at four different time points in the presence of M-CSF and RANKL (Figure 2C). The results showed that GSPE completely inhibited osteoclast formation on day 0–1 (98.6%) of GSPE exposure after RANKL treatment. A similar inhibitory effect was observed when GSPE was added on day 1–2 (88.1%) or day 2–3 (81.2%). However, when added on day 3–4, there was a decrease in the inhibitory effect of GSPE (67.5%) (Figure 2D). These findings suggest that GSPE displays its inhibitory effect from the beginning of osteoclast differentiation. We then examined whether GSPE inhibits the bone resorption function of osteoclasts. As shown in Figure 2E,F, in the control, numerous resorption pits were produced by mature osteoclasts cultured on top of HA-coated plates or dentin slices, while these resorption pits were significantly lower in case of GSPE treatment.

### 3.3. GSPE Downregulates RANKL-Induced Expression of c-Fos, NFATc1, and Osteoclastogenic Marker Genes

**Figure 3:**
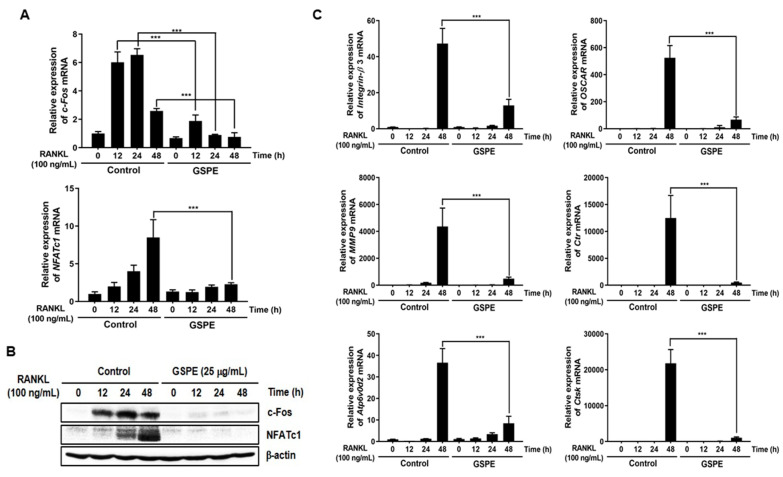
GSPE downregulates RANKL-induced c-Fos, NFATc1, and osteoclastogenic-specific marker genes. BMMs were stimulated with 30 ng/mL M-CSF and 100 ng/mL RANKL in the presence or absence of 25 μg/mL GSPE for the indicated time periods. (**A**) mRNA expression levels of the *c-Fos* and *NFATC1* genes were evaluated using real-time RT-PCR. (**B**) The protein expression levels of c-Fos and NFATc1 were evaluated using Western blot analysis. (**C**) The expression levels of *Integrin-β3*, *OSCAR*, *MMP9*, *Ctr*, *Atp6v0d2*, and *CtsK* mRNA were analyzed using real-time RT-PCR. *** *p* < 0.001 versus control. Abbreviations: GSPE, grape seed proanthocyanidin extract; RANKL, receptor activator of nuclear factor kappa-Β ligand.

To assess whether GSPE has any effect on the induction of c-Fos, NFATc1, and osteoclast-specific genes, the corresponding mRNA and protein levels were examined using qRT-PCR and Western blot analysis. As shown in Figure 3A, GSPE inhibited the RANKL-induced expression of *c-Fos* and *NFATC1* mRNA. Corresponding to the mRNA results, there was an increase in the protein levels of c-Fos and NFATc1 in response to RANKL; this increase was significantly inhibited by GSPE (Figure 3B). In addition, GSPE also suppressed the RANKL-induced increase in the mRNA expression of *Integrin-β_3_*, *OSCAR*, *MMP9*, *Ctr*, *Atp6v0d2*, and *CtsK* at 48 h (Figure 3C). These results demonstrate that GSPE inhibited the RANKL-induced osteoclastogenesis through the downregulation of expression of transcription factors, such as c-Fos and NFATc1 and other osteoclast-specific genes.

### 3.4. GSPE Suppresses Osteoclastogenesis by Inhibition of RANKL-Induced NF-κB Signaling

**Figure 4:**
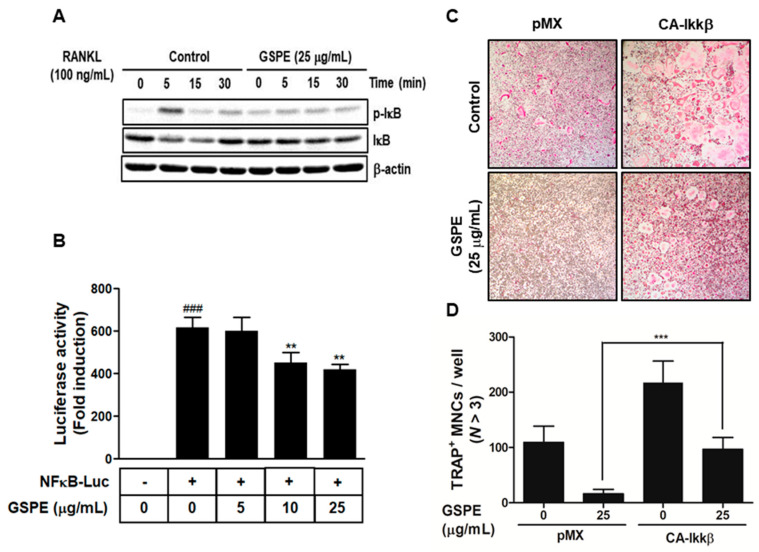
GSPE suppresses osteoclast differentiation by downregulating the NF-κB signaling pathway. (**A**) Bone marrow macrophages (BMMs) were pretreated with 25 μg/mL GSPE for 1 h in the presence of 10 ng/mL M-CSF, followed by stimulation with 100 ng/mL RANKL for the indicated time period. Western blot analysis of the whole-cell lysates was performed using phospho-IκB and IκB antibodies. β-actin served as the internal control. (**B**) HEK293R cells were co-transfected with an NF-κB luciferase reporter and β-galactosidase to normalize the transfection efficiency. The transfected cells were incubated for 24 h in the presence of the indicated concentrations of GSPE. Luciferase activity has been expressed as the fold induction relative to the corresponding negative control condition. ^###^ *p* < 0.001 versus negative control; ** *p* < 0.01 versus vehicle. (**C**) BMMs were infected with pMX-constitutively active (CA)-Iκκβ-IRES-EGFP. Infected BMMs were pretreated with or without 25 μg/mL GSPE in the presence of 30 ng/mL M-CSF for 24 h, followed by stimulation with 100 ng/mL RANKL. After 4 days, osteoclasts were detected using TRAP staining and (**D**) TRAP-positive MNCs were counted. *** *p* < 0.001 versus control. Abbreviations: GSPE, grape seed proanthocyanidin extract; RANKL, receptor activator of nuclear factor kappa-Β ligand; MNCs, multinucleated cells; NF-κB-LUC, nuclear factor-κB luciferase.

To identify the mechanism involved in the inhibition of osteoclastogenesis by GSPE, we examined IκB and p-IκB, which are known to play crucial roles in osteoclast differentiation. As shown in Figure 4A, RANKL-stimulated phosphorylation of IκB at 5 min was inhibited by GSPE, and the degradation of IκB induced by RANKL at 5 min and 15 min was recovered by GSPE. Additionally, the NF-κB luciferase reporter displayed a significantly high luciferase activity that was considerably inhibited by GSPE in a dose-dependent manner (Figure 4B). Because GSPE inhibits the phosphorylation and degradation of IκB, we then checked whether GSPE alters the IκB kinase (Ikk) signal, which leads to IκB phosphorylation. We used a retrovirus to overexpress the constitutively active form of Ikkβ (CA-Ikkβ) in BMMs. As shown in Figure 4C, transfection of CA-Ikkβ resulted in a partial reversion of the GSPE-mediated inhibition of osteoclast differentiation. The number of reversed osteoclasts was also counted (Figure 4D). These results suggest that GSPE exerted an inhibitory effect on osteoclast differentiation by regulating the NF-κB signaling pathway.

### 3.5. GSPE Leads to the Phosphorylation of Downstream Signaling Molecules of the Akt and MAPK Pathways, such as p38, JNK, and ERK, without RANKL Stimulation

**Figure 5:**
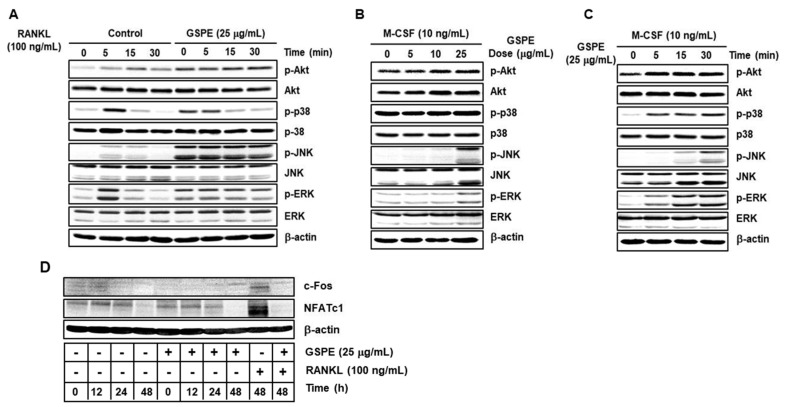
GSPE promotes the phosphorylation of components of the Akt and MAPK signaling pathways, including p38, JNK, and ERK. (**A**) Bone marrow macrophages (BMMs) were pretreated with 25 μg/mL GSPE for 1 h in the presence of 10 ng/mL M-CSF, followed by stimulation with 100 ng/mL RANKL for the indicated time period. Western blot analysis of the whole-cell lysates was performed using the indicated antibodies. β-actin served as the internal control. (**B**) BMMs in the presence of 10 ng/mL M-CSF were stimulated with the indicated doses of GSPE for 15 min. (**C**) BMMs in the presence of 10 ng/mL M-CSF were stimulated with 25 μg/mL GSPE for the indicated time period. (**D**) BMMs were stimulated with 25 μg/mL GSPE in the indicated conditions. Co-stimulation with 100 ng/mL RANKL was performed for comparison. Whole-cell lysates were analyzed by performing Western blotting with the indicated antibodies. Abbreviations: GSPE, grape seed proanthocyanidin extract; RANKL, receptor activator of nuclear factor kappa-Β ligand; c-Fos, cellular Fos; NFATc1, nuclear factor of activated T cells.

Activation of the Akt and MAPK signaling pathways is important for the proliferation and differentiation of osteoclasts. To examine the effect of GSPE on these signaling pathways, osteoclast precursors were stimulated with RANKL in the presence or absence of GSPE for 0 to 30 min. Contrary to expectations, the phosphorylation of components of the MAPK and Akt pathways was not inhibited post-GSPE treatment, but rather enhanced signals were detected (Figure 5A, Figure S2A). To identify whether these enhanced signals were related to RANKL stimulation, BMMs were stimulated with GSPE without RANKL stimulation in a dose- and time-dependent manner. Interestingly, GSPE stimulation induced phosphorylation of components of the MAPK and Akt signaling pathways (especially JNK and ERK signals) independently, in both a dose-and time-dependent manner in the presence of M-CSF without RANKL stimulation (Figure 5B,C, Figure S2B,C). Since GSPE was found to activate MAPK and Akt pathways, we then confirmed the activity of c-Fos and NFATc1, the major transcription factors involved in osteoclast differentiation, to confirm whether GSPE has a similar effect as RANKL in osteoclasts. The expression levels of c-Fos and NFATc1 did not change in the presence or absence of GSPE without stimulation of RANKL. As expected, the protein levels of c-Fos and NFATc1 in the RANKL-stimulated sample, as well as the positive control, increased appropriately in response to RANKL (Figure 5D).

### 3.6. GSPE Promotes Proliferation of Osteoclast Precursor Cells and Inhibits Apoptosis in the Presence of RANKL

**Figure 6:**
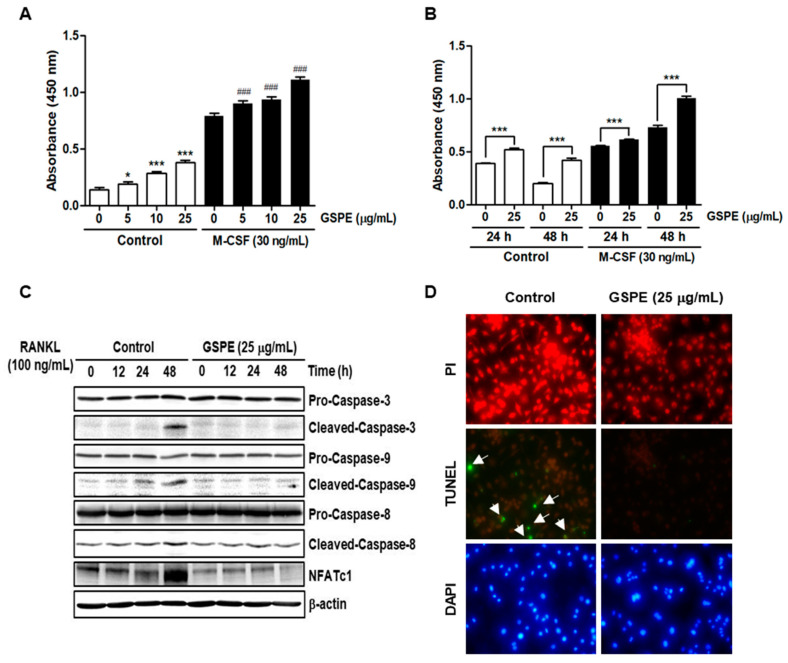
GSPE promotes proliferation and inhibits apoptosis. (**A**) BMMs were cultured for 48 h with the indicated doses of GSPE in the presence or absence of 30 ng/mL M-CSF. * *p* < 0.05; *** *p* < 0.001 versus 0 g/mL in control, ^###^ *p* < 0.001 versus each dose in control. (**B**) BMMs were cultured in the presence or absence of 25 μg/mL GSPE and 30 ng/mL M-CSF for the indicated time periods. Cell viability was determined using XTT assay. *** *p* < 0.001. (**C**) BMMs were stimulated using 100 ng/mL RANKL in the presence or absence of 25 μg/mL GSPE with 30 ng/mL M-CSF for the indicated time periods. The protein expression levels of caspase-3, -8, and -9 were evaluated using Western blot analysis. (**D**) BMMs were cultured with 30 ng/mL M-CSF and 100 ng/mL RANKL in the presence or absence of 25 μg/mL GSPE. The cells were fixed, permeabilized, and stained with TUNEL, PI, and DAPI and then examined under a fluorescence microscope. Abbreviations: GSPE, grape seed proanthocyanidin extract; RANKL, receptor activator of nuclear factor kappa-Β ligand; M-CSF, macrophage colony-stimulating factor; TUNEL, Terminal deoxynucleotidyl transferase dUTP nick end labeling; PI, propidium iodide; DAPI, 4′,6-Diamidino-2-phenylindole dihydrochloride.

Because activation of Akt and MAPK pathways is known for enhancing the proliferation and survival of osteoclast precursors, we tested the effect of GSPE on the proliferation of osteoclast precursors in the presence of M-CSF. Regardless of the presence or absence of M-CSF, the proliferation of osteoclast precursor cells increased with increasing doses of GSPE (Figure 6A). This was also confirmed at two different time points, 24 and 48 h, in osteoclast precursors treated with 25 μg/mL GSPE (Figure 6B). Next, we examined the effect of GSPE on the apoptosis of osteoclasts stimulated by RANKL. As shown in Figure 6C and Figure S3, 48 h after RANKL treatment, there was an increase in the levels of cleaved caspase-3, -8, and -9; this increase in the levels of cleaved forms of caspases was inhibited by GSPE. Corresponding to this result, the TUNEL-stained cells that were observed in the case of the RANKL-treated cells were not observed in the presence of GSPE (Figure 6D).

## 4. Discussion

GSPE has various pharmacological activities including anti-inflammatory, anti-oxidative, anti-bacterial, anti-viral, vasodilatory, anti-allergic, cardioprotective, and anti-carcinogenic activities [14,15,16,17]. In particular, GSPE has been reported to have a protective and therapeutic effect on osteoporosis [22], bone necrosis [23], and inflammatory autoimmune arthritis [18,19,20]; it has also been shown to increase bone density and strength [24,25]. Based on this evidence, we tried to elucidate the role and mechanism of osteoclasts in the protective effect of GSPE in bone loss. In this study, we determined the protective effect of GSPE on LPS-induced inflammatory bone loss in vivo. In addition, we investigated the effects of GSPE on osteoclast proliferation, differentiation, apoptosis, and bone resorbing function, and its underlying signaling mechanisms.

Since osteoclasts have a short lifespan, any regulation that prolongs their viability can increase osteoclast activity [26]. The number and activity of osteoclast is determined by the ability to proliferate, survive, and differentiate osteoclast precursors [27]. M-CSF acts primarily to promote the proliferation and survival of osteoclast precursors during the osteoclastogenesis process [28]. RANKL acts as a major factor in the differentiation of osteoclast precursors into mature osteoclasts [28]. Our data suggest that GSPE strongly inhibited RANKL-induced osteoclast differentiation, especially when added in the early stage of culture (Figure 2A–D). In addition, GSPE attenuated mature osteoclastic bone resorption (Figure 2E,F). Osteoclastogenesis is a multi-step process involving commitment, fusion, and activation [29,30]. Osteoclast differentiation is mainly triggered by RANKL and RANK on the osteoclast precursor and amplified by downstream transduction signaling. In particular, NFATc1 is a master transcription factor of osteoclast differentiation that promotes the expression of osteoclast-specific genes. NFATc1 play an important role in the expression of osteoclast-specific genes, such as *Integrin-β_3_*, *OSCAR*, *MMP9*, *Ctr*, *Atp6v0d2*, and *CtsK*, which are required for osteoclast differentiation, fusion, and function. In our study, GSPE was found to inhibit the mRNA and protein expression of c-Fos and NFATc1 (Figure 3A,B). Also, the inhibitory effect of GSPE via downregulation of c-Fos and NFATc1 was confirmed by changes in the mRNA expression levels of osteoclast-specific genes (Figure 3C). The binding of RANK to RANKL activates NF-κB, which is one of the most important transcription factors in osteoclast differentiation [11,12,27]. IκB binds to NF-κB, which prevents it from translocating to the nucleus and phosphorylation by the Ikk complex [31]. Subsequently, the ubiquitination and proteasomal degradation of IκB induces NF-κB to translocate to the nucleus and stimulate the transcription of the target gene [31]. In this study, GSPE was found to inhibit the phosphorylation and degradation of IκB (Figure 4A), while Ikkβ overexpression partially reversed the GSPE-induced inhibition of osteoclastogenesis (Figure 4C,D). Moreover, the luciferase assay confirmed that GSPE suppresses NF-κB transcription activity (Figure 4B). These results indicate that GSPE inhibited the RANKL-induced transcription activity of NF-κB through increased phosphorylation and degradation of IκB by Ikkβ.

Akt and MAPK signaling pathways, including downstream signaling molecules p38, JNK, and ERK, are important for the regulation of osteoclastogenesis [9,10]. Contrary to expectations, our study showed that GSPE further activates RANKL-induced phosphorylation of Akt, p38, JNK, and ERK (Figure 5A). Moreover, GSPE was found to increase the phosphorylation of Akt, p38, JNK, and ERK in a dose- or time-dependent manner in the presence of M-CSF, without RANKL stimulation (Figure 5B,C). Therefore, we tested whether GSPE plays a role similar to RANKL; we found that GSPE did not induce c-Fos and NFATc1 activity by GSPE treatment without RANKL stimulation (Figure 5D). Akt and MAPK signaling pathways play an important role not only in osteoclast differentiation, but also in the regulation of proliferation and apoptosis. It has been reported that M-CSF-activated Akt and MAPK signaling pathways are primarily involved in the proliferation and survival of osteoclast precursor, whereas RANKL-induced Akt and MAPK activation is mainly associated with osteoclast differentiation [9]. However, in this study, it was confirmed that RANKL-induced Akt and MAPK activation in the presence of GSPE preferentially affects the pathways that promote osteoclast proliferation, while suppressing apoptosis rather than osteoclast differentiation (Figure 6).

In keeping with its anti-osteoclastogenic effect in vitro, GSPE prevented LPS-induced inflammatory bone loss in vivo. LPS injection leads to inflammatory bone loss by recruiting inflammatory cells, increasing the number of osteoclasts, and activating osteoclast resorption. Micro-CT analysis performed in this study indicated that GSPE treatment for LPS-injected mice increased BV/TV, Tb.Th, and Tb.N, indicating that GSPE restored LPS-induced bone loss. Bone histomorphometry analysis showed that GSPE significantly reduced LPS-induced bone erosion and TRAP-positive osteoclast formation in vivo. Despite these promising results, our study has some limitations. Inflammatory bone loss caused by LPS in vivo is induced by a complex process of many cells, including osteoblasts and osteoclasts. In this study, the effect of GSPE on osteoclast proliferation, differentiation, survival and bone resorption was investigated. However, we cannot rule out the possibility that GSPE will affect osteoblast bone formation. Therefore, further research is needed to evaluate the effect of GSPE on osteoblast bone formation. Also, our study focused only on pathological bone loss due to LPS, which is dominated by excessive osteoclast activity. Therefore, to fully elucidate the effects and mechanisms of action of GSPE in bone remodeling, further studies are needed to evaluate the effects of GSPE on normal bone or other pathological conditions.

## 5. Conclusions

Our findings show that GSPE protects against LPS-induced inflammatory bone loss in vivo. In vitro mechanism studies demonstrate that not only does GSPE inhibit osteoclast differentiation but it also inhibits apoptosis and promotes proliferation. Our results extend the understanding of the role of GSPE in bone metabolism and suggest the potential therapeutic application of GSPE against osteoclast-mediated destructive diseases.

## Figures and Tables

**Table 1:** Primer sequences used for real-time PCR analysis.

Gene	Forward (5′ → 3′)	Reverse (5′ → 3′)	Product (bp)
*glyceraldehyde-3-phosphate dehydrogenase (GAPDH)*	TCAAGAAGGTGGTGAAGCAG	AGTGGGAGTTGCTGTTGAAGT	102
*proto-oncogene cellular Fos* (*c-Fos*)	GGTGAAGACCGTGTCAGGAG	TATTCCGTTCCCTTCGGATT	110
*nuclear factor of activated T cells (NFATC1)*	GAGTACACCTTCCAGCACCTT	TATGATGTCGGGGAAAGAGA	110
*Integrin*-*β_3_*	GGAGTGGCTGATCCAGATGT	TCTGACCATCTTCCCTGTCC	138
*osteoclast-associated receptor (OSCAR)*	GGAATGGTCCTCATCTCCTT	TCCAGGCAGTCTCTTCAGTTT	112
*Matrix metallopeptidase 9 (MMP9)*	TCCAACCTCACGGACACCC	AGCAAAGCCGGCCGTAGA	107
*Calcitonin receptor (Ctr)*	TCCAACAAGGTGCTTGGGAA	CTTGAACTGCGTCCACTGGC	141
*ATPase H+ Transporting V0 Subunit D2 (Atp6v0d2)*	GACCCTGTGGCACTTTTTGT	GTGTTTGAGCTTGGGGAGAA	102
*cathepsin K (CtsK)*	CCAGTGGGAGCTATGGAAGA	CTCCAGGTTATGGGCAGAGA	118

## Supplementary Materials

The following are available online at https://www.mdpi.com/2072-6643/12/10/3164/s1, Figure S1: BMMs were cultured for 3 days at the indicated doses of GSPE in the presence of M-CSF (30 ng/mL). Cytotoxicity was determined by XTT assay. Figure S2: The graph indicates the phosphoprotein-to-total protein rations of each molecule in Figure 5A, Figure 5B, and Figure 5C. Figure S3: The graph indicates density of the cleaved-caspase-3, -9, and -8 bands.

## References

[1] Feng X., McDonald J.M. (2011). Disorders of bone remodeling. Annu. Rev. Pathol..

[2] McLean R.R. (2009). Proinflammatory cytokines and osteoporosis. Curr. Osteoporos. Rep..

[3] Tanaka Y., Nakayamada S., Okada Y. (2005). Osteoblasts and osteoclasts in bone remodeling and inflammation. Curr. Drug Targets Inflamm. Allergy.

[4] Pereira M., Petretto E., Gordon S., Bassett J.H.D., Williams G.R., Behmoaras J. (2018). Common signaling pathways in macrophage and osteoclast multinucleation. J. Cell Sci..

[5] Roodman G.D. (2006). Regulation of osteoclast differentiation. Ann. N. Y. Acad. Sci..

[6] Väänänen K. (2005). Mechanism of osteoclast mediated bone resorption-rationale for the design of new therapeutics. Adv. Drug Deliv. Rev..

[7] Lee A.W., States D.J. (2000). Both src-dependent and-independent mechanisms mediate phosphatidylinositol 3-kinase regulation of colony-stimulating factor 1-activated mitogen-activated protein kinases in myeloid progenitors. Mol. Cell Biol..

[8] Takeshita S., Faccio R., Chappel J., Zheng L., Feng X., Weber J.D., Teitebaum S.L., Ross F.P. (2007). c-Fms tyrosine 559 is a major mediator of M-CSF-induced proliferation of primary macrophages. J. Biol. Chem..

[9] Kim J.H., Kim N. (2016). Signaling pathway in osteoclast differentiation. Chonnam Med. J..

[10] Ikeda F., Matsubara T., Tsurukai T., Hata K., Nishimura R., Yoneda T. (2008). JNK/c-Jun signaling mediates an anti-apoptotic effect of RANKL in osteoclasts. J. Bone Miner. Res..

[11] Boyce B.F., Xiu Y., Li J., Xing L., Yao Z. (2015). NF-κB-mediated regulation of osteoclastogenesis. Endocrinol. Metab. (Seoul).

[12] Novack D.V., Yin L., Hagen-Stapleton A., Schreiber R.D., Goeddel D.V., Ross F.P., Teitelbaum S.L. (2003). The IκB function of NF-κB2 p100 controls stimulated osteoclastogenesis. J. Exp. Med..

[13] Takayanagi H., Kim S., Koga T., Nishina H., Isshiki M., Yoshida H., Saiura A., Isobe M., Yokochi T., Inoue J. (2002). Induction and activation of the transcription factor NFATc1 (NFAT2) integrate RANKL signaling in terminal differentiation of osteoclasts. Dev. Cell.

[14] Henriet J.P. (1993). Veno-lymphatic insufficiency. 4729 patients undergoing hormonal and procyanidol oligomer therapy. Phlebologie.

[15] Ma Z.F., Zhang H. (2017). Phytochemical constituents, health benefits, and industrial applications of grape seeds: A mini-review. Antioxidnats (Basel).

[16] Bagchi D., Swaroop A., Preuss H.G., Bagchi M. (2014). Free radical scavenging, antioxidant and cancer chemoprevention by grape seed proanthocyanidin: An overview. Mutat. Res..

[17] Li W.G., Zhang X.Y., Wu Y.J., Tian X. (2001). Anti-inflammatory effect and mechanism of proanthocyanidins from grape seeds. Acta Pharmacol. Sin..

[18] Cho M.L., Heo Y.J., Park M.K., Oh H.J., Park J.S., Woo Y.J., Ju J.H., Oark S.H., Kim H.Y., Min J.K. (2009). Grape seed proanthocyanidin extract (GSPE) attenuates collagen-induced arthritis. Immunol. Lett..

[19] Jhun J.Y., Moon S.J., Yoon B.Y., Byun J.K., Kim E.K., Yang E.J., Ju J.H., Hong Y.S., Min J.K., Park S.H. (2013). Regulation of STAT3 proteins contributes to treg differentiation and attenuates inflammation in a murine model of obesity-associated arthritis. PLoS ONE.

[20] Woo Y.J., Joo Y.B., Jung Y.O., Ju J.H., Cho M.L., Oh H.J., Jhun J.Y., Park M.K., Park J.S., Kang C.M. (2011). Grape seed proanthocyanidin extract ameliorates monosodium iodoacetate-induced osteoarthritis. Exp. Mol. Med..

[21] Livak K.J., Schmittgen T.D. (2001). Analysis of relative gene expression data using real-time quantitative PCR and the 2(-Delta Delta C(T)) method. Methods.

[22] Xhu W., Yin Z., Zhang Q., Guo S., Shen Y., Liu T., Liu B., Wan L., Li S., Chen X. (2019). Proanthocyanidins inhibit osteoclast formation and function by inhibiting the NF-κB and JNK signaling pathways during osteoporosis treatment. Biochem. Biophys. Res. Commun..

[23] Song Q., Shi Z., Bi W., Liu R., Zhang C., Wang K., Dang X. (2015). Beneficial effect of grape seed proanthocyanidin extract in rabbits with steroid-induced osteonecrosis via protecting against oxidative stress and apoptosis. J. Orthop. Sci..

[24] Tofani L., Maki K., Kojima K., Kimura M. (2004). Beneficial effects of grape seed proanthocyanidins extract on formation of tibia bone in low-calcium feeding rats. Pediatr. Dent..

[25] Yahara N., Tofani I., Maki K., Kojima K., Kojima Y., Kimura M. (2005). Mechanical assessment of effects of grape seed proanthocyanidins extract on tibial bone diaphysis in rats. J. Musculoskelet. Neuronal Interact..

[26] Soysa N.S., Alles N. (2019). Positive and negative regulators of osteoclast apoptosis. Bone Rep..

[27] Lerner U.H. (2019). Osteoclasts in health and disease. Pediatr. Endocrinol. Rev..

[28] Feng X., Teitelbaum S.L. (2013). Osteoclasts: New insights. Bone Res..

[29] Boyce B.F. (2013). Advances in the regulation of osteoclasts and osteoclast functions. J. Dent. Res..

[30] Boyle W.J., Simonet W.S., Lacey D.L. (2003). Osteoclast differentiation and activation. Nature.

[31] Huang W.C., Hung M.C. (2013). Beyond NF-kB activation: Nuclear functions of IkB kinase α. J. Biomed. Sci..

