# Supplementary material for: Grape Seed Proanthocyanidin Extract Prevents Bone Loss via Regulation of Osteoclast Differentiation, Apoptosis, and Proliferation
Source: Nutrients. 2020 Oct 16;12(10):3164. doi: 10.3390/nu12103164 (PMC7602819; doi:10.3390/nu12103164)
Supplement: Supplementary file 1 [file nutrients-12-03164-s001.pdf]

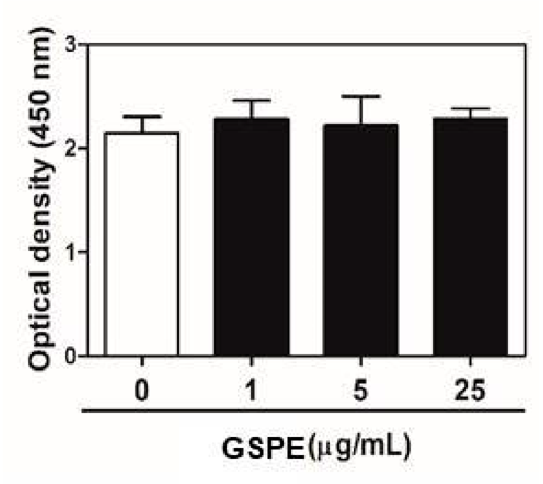

Figure S1. BMMs were cultured for 3 days at the indicated doses of GSPE in the presence of M-CSF (30 ng/mL). Cytotoxicity was determined by XTT assay.

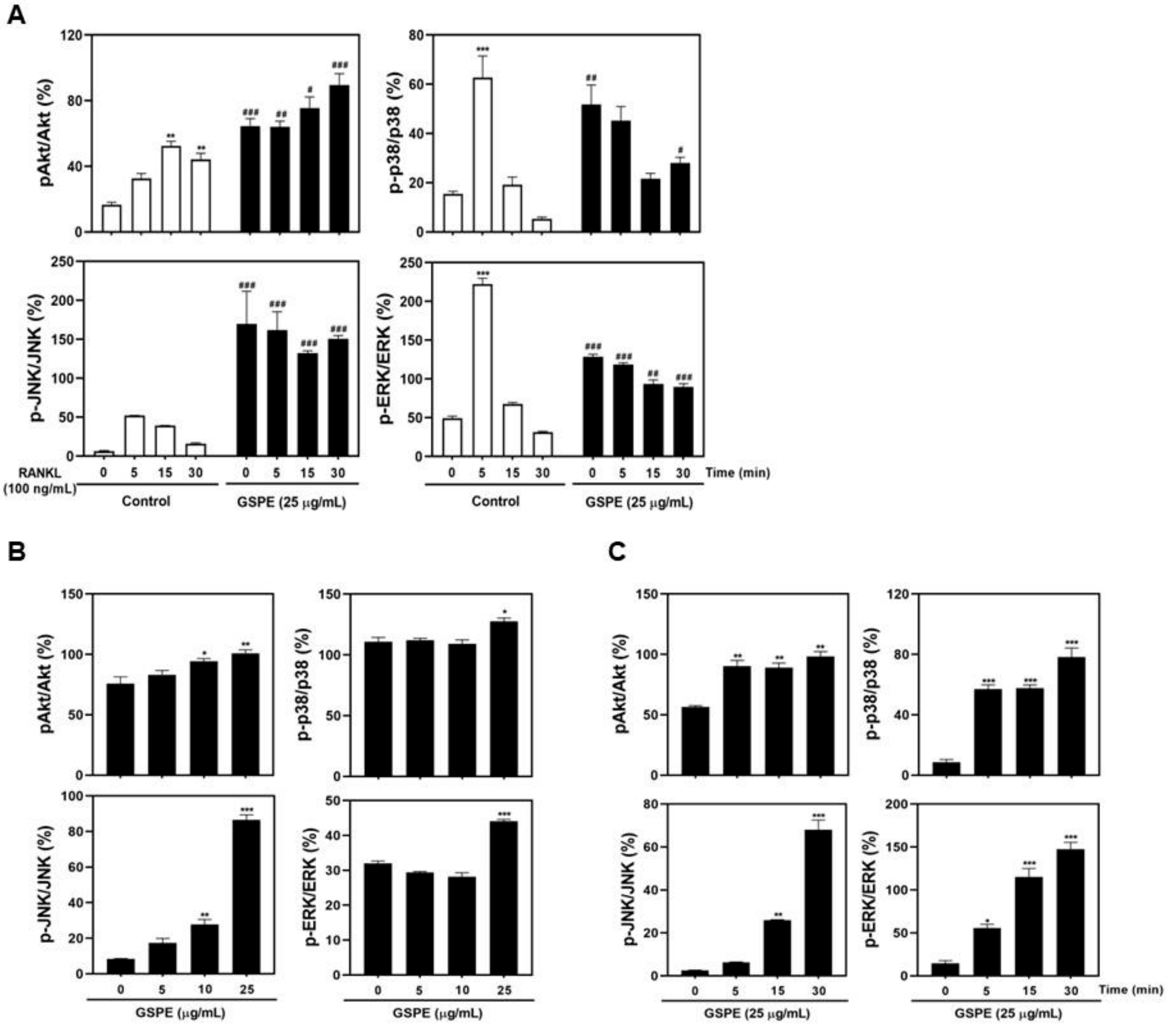

Figure S2. The graph indicates the phosphoprotein-to-total protein rations of each molecule in Figure 5A, B, and C. Quantification of relative ratio of band intensity was performed using Image J software. The phosphoprotein-to-total protein ratios (%) are represented as the mean ± standard deviation. $^{**}p$

< 0.01, $^{***}p < 0.001$ versus Control at 0 min or 0 μg/mL; $^{\#}p < 0.05$, $^{\#\#}p < 0.01$, $^{\#\#\#}p < 0.001$ versus Control at each time.

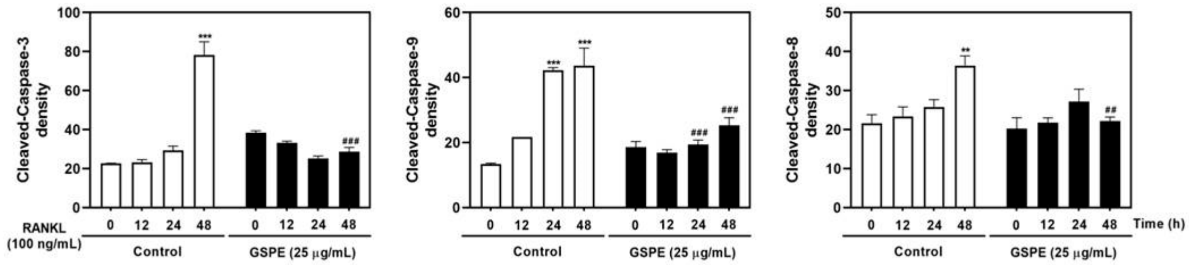

Figure S3. The graph indicates density of the cleaved-caspase-3, -9, and -8 bands. Quantification of band intensity was performed using Image J software. The band intensity is represented as the mean ± standard deviation. $^{**}p < 0.01$, $^{***}p < 0.001$ versus Control at 0 h; $^{\#\#}p < 0.01$, $^{\#\#\#}p < 0.001$ versus Control at each time.
